# Application of double-negative T cells in haematological malignancies: recent progress and future directions

**DOI:** 10.1186/s40364-022-00360-w

**Published:** 2022-03-14

**Authors:** Xingchi Chen, Dongyao Wang, Xiaoyu Zhu

**Affiliations:** 1 Department of hematology, the First Affiliated Hospital of USTC, Division of Life Sciences and Medicine, University of Science and Technology of China, Hefei, 230001 Anhui China; 2 Blood and Cell Therapy Institute, Division of Life Sciences and Medicine, University of Science and Technology of China, Hefei, 230001 Anhui China; 3 Anhui Provincial Key Laboratory of Blood Research and Applications, Hefei, 230001 Anhui China

**Keywords:** DNT cells, Hematologic malignancies, ACT, GVHD, Allo-HSCT

## Abstract

Haematologic malignancies account for a large proportion of cancers worldwide. The high occurrence and mortality of haematologic malignancies create a heavy social burden. Allogeneic haematopoietic stem cell transplantation is widely used in the treatment of haematologic malignancies. However, graft-versus-host disease and relapse after allogeneic haematopoietic stem cell transplantation are inevitable. An emerging treatment method, adoptive cellular therapy, has been effectively used in the treatment of haematologic malignancies. T cells, natural killer (NK) cells and tumour-infiltrating lymphocytes (TILs) all have great potential in therapeutic applications, and chimeric antigen receptor T (CAR-T) cell therapy especially has potential, but cytokine release syndrome and off-target effects are common. Efficient anticancer measures are urgently needed. In recent years, double-negative T cells (CD3^+^CD4^−^CD8^−^) have been found to have great potential in preventing allograft/xenograft rejection and inhibiting graft-versus-host disease. They also have substantial ability to kill various cell lines derived from haematologic malignancies in an MHC-unrestricted manner. In addition, healthy donor expanded double-negative T cells retain their antitumour abilities and ability to inhibit graft-versus-host disease after cryopreservation under good manufacturing practice (GMP) conditions, indicating that double-negative T cells may be able to be used as an off-the-shelf product. In this review, we shed light on the potential therapeutic ability of double-negative T cells in treating haematologic malignancies. We hope to exploit these cells as a novel therapy for haematologic malignancies.

## Background

Haematologic malignancies are cancers driven by genetic or epigenetic lesions in haematopoietic stem cells (HSCs) that lead to the development of cancer stem cells, and haematologic malignancies result in fewer functional haematologic cells and invasion into peripheral lymph organs [1, 2]. There are many types of haematologic malignancies, such as leukaemia, lymphoma, and myeloma. The global cancer statistics in 2021 produced by the International Agency for Research on Cancer showed a high incidence and death rate of haematologic malignancies, of which leukaemia ranked in the top ten in terms of mortality (the mortality of leukemia is 3.1%) [3]. Moreover, the latest cancer statistics published by Siegel et al. showed leukaemia was the most common childhood cancer, accounting for 28% of cases [4]. The high occurrence and mortality of haematologic malignancies indicate the inefficiency of therapy and poor prognosis, implying the urgent need for novel treatment methods.

Allogeneic haematopoietic stem cell transplantation (allo-HSCT) plays a crucial role in various haematologic malignancies. It usually begins with high doses of chemotherapy and radiation, followed by infusion of donor-specific haematopoietic stem cells derived from peripheral blood, bone marrow, or umbilical cord blood. The constitution of a new haematologic system is significantly related to the outcome. Graft-versus-host disease (GVHD) and relapse are two main causes of death after allo-HSCT [5]. Adoptive cellular therapy (ACT) is an emerging method in the treatment of haematologic cancers, involving the transfusion of cells with great antitumour capacity [6]. Many kinds of cells have been used in ACT and have demonstrated to be effective in the treatment of haematologic cancers, such as chimeric antigen receptor T (CAR-T) cells [7], chimeric antigen receptor natural killer (CAR-NK) cells [8], natural killer (NK) cells [9], and tumour-infiltrating-lymphocytes (TILs) [10], but CAR-T cell therapy is always accompanied by a series of side effects, such as serious cytokine release syndrome (CRS) and immune effector cell associated Neurotoxicity syndrome (ICANS) [11]. However, a newly identified type of cell, double-negative T cells (DNTs), have been found to be effective effector cells for ACT.

**Fig. 1:**
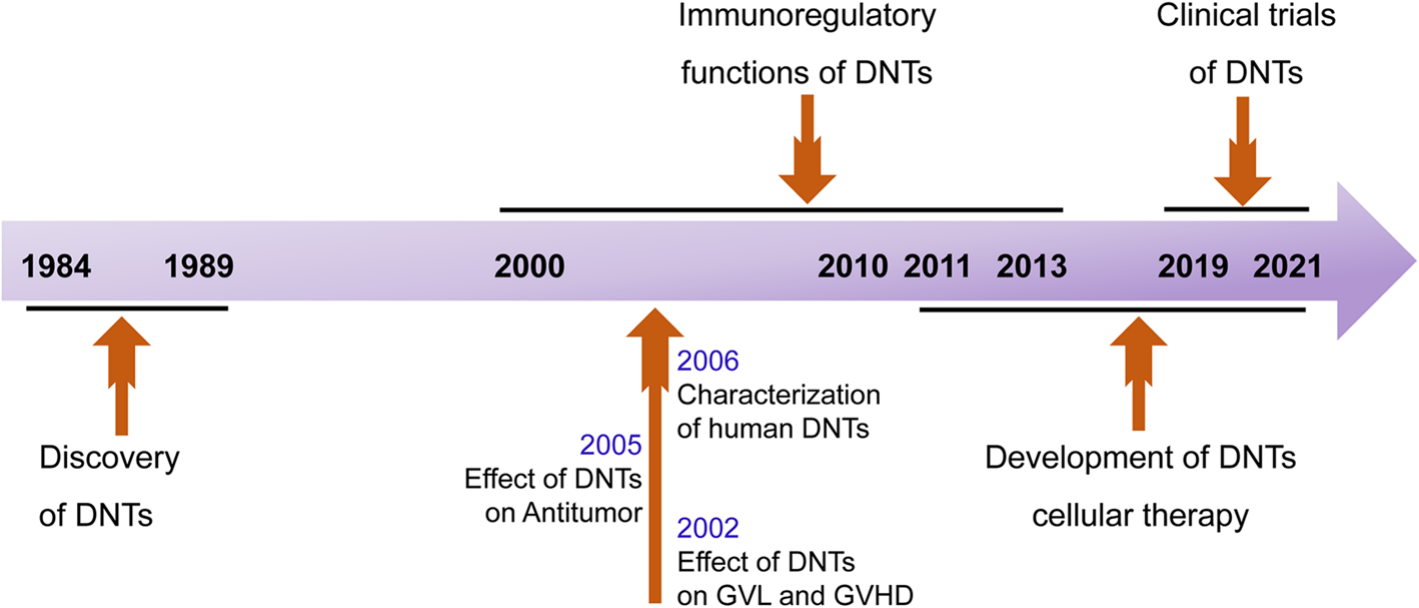
The timeline of studies in development of DNTs function in immunoregulation and anticancer. Since the discovery of DNTs in 1984, a lot of research have been performed in DNTs development and function. DNTs, double negative T cells; GVHD, graft-versus-host disease; GVL, graft-versus-leukaemia

DNTs, also called natural suppressor (NS) cells, were first described by Strober for their suppressive activity and “null phenotype” without antigen specificity [12] (Fig. 1). Subsequently, they demonstrated inhibition of GVHD without major histocompatibility complex (MHC) restriction [13] and were identified to express the cell surface makers αβ-T cell receptor (TCR) and CD3, but without CD4 and CD8 [14]. In 2000, Zhang et al. [15] proposed DNTs for the first time, and these DNTs were characterized as abnormal regulatory T cells expressing αβ-TCR and CD25 but without CD4 or CD8 expression. Although DNTs account for only 1% ~ 5% of peripheral lymphocytes [16], many researchers have demonstrated the strong suppressive ability of DNTs towards CD8^+^ T cells, CD4^+^ T cells, B cells and NK cells in vitro and in vivo, which leads to xenograft or allograft transplantation tolerance and powerful prevention of GVHD [17–19]. In addition, DNTs possess strong antitumour characteristics without MHC restriction in vitro and in vivo and can be efficiently expanded ex vivo [20]. Therefore, we suggest that DNTs have great potential as off-the-shelf products and may have superior performance in the ACT field. Here, we review the functions and clinical application of DNTs to aid understanding.

## Development and subgroups of DNTs

Maturation of most T cells begins with migration of bone marrow-derived progenitor cells to the thymus, where they go through T cell receptor (TCR) rearrangement, positive selection and negative selection to acquire diverse TCR repertoires and tolerance to autoantigens [21]. However, DNTs, considered unconventional T cells, have controversial origins and maturation pathways due to their heterogeneous cell surface marker expression and differences in cytokine secretion [22]. Human T cells can mature into αβT cells and γδT cells, and the signal strength of the TCR plays a critical role in lineage differentiation [23]. The quality and magnitude of CD8^+^T-cell responses were important to overcome virus infections and kill tumour cells [24]. In addition, naïve CD4^+^ T cells could differentiate into effector Th cells that are characterized by the expression of distinct cytokines and transcription factors. These cells have differential immune functions [25–28]. What is more, in Hayes’s study, DN3 stage (a later double-negative (DN) stage in thymic T cell development) T cells converted into DNTs through strong signal transduction of the pre-TCR/CD3 complex [29]. Leonid and colleagues proposed that under TCR signalling, double-positive (DP) thymocytes are prevented from polarizing into conventional single-positive (SP) T cells, instead undergoing developmental diversion and then becoming DNTs. Subsequently, these DNTs could migrate to the intestine and differentiate into intraepithelial lymphocytes (IELs). Here, the features and properties of T cell subsets were summarized and shown in the form of table (Table 1).

**Table 1:** The features and properties of T cell subsets

T cell subsets	Distinct transcription factor	Distinct cytokines	Immune functions	References
Th1	T-bet	IFN-γ	Participate in cellular immunity against intracellular microorganisms	[25–28]
Th2	GATA-3	IL-4, IL-5, IL-13	Control helminths and other extracellular pathogens	[25–28]
Th9	STAT6, IRF4	IL-9	Enhance allergic inflammation and airway hyperreactivity; promote tumour progression or anti-tumour immunity	[25–28]
Th17	RORγt	IL-17a, IL-17f, IL-21, IL-22	Clear extracellular bacteria and fungi	[25–28]
Tfh	Bcl-6	IL-4, IL-21	Regulate the maturation of B cell responses	[25–28]
Treg	Foxp3	IL-10, TGF-β	Participate in maintenance of peripheral tolerance and prevention of autoimmunity	[25–28]
DNT	IKZF2	IFN-γ, Granzyme B	Participate in regulation of adaptive and innate immunity; anti-tumour	[30]
CD8^+^ T		IFN-γ, TNF-α, IL-2	Clear virus and tumour cells	[24]

*Th* T helper, *IFN* Interferon, *IL* Interleukin, *Tfh* Follicular helper T, *Treg* Regulatory T cell, *DNT* Double negative T, *TNF* tumor necrosis factor

Several studies have identified that activated peripheral CD4^+^ or CD8^+^ T cells can transform into DNTs, in the presence of different cytokines, such as IL-2 [31–35]. Fas-deficient or Fas ligand-deficient mice, which exhibit accumulation of DNTs in secondary lymphoid organs [36], are widely used in DNT research. Some researchers have proposed that the accumulation of DNTs in lpr mice results from Fas pathway deficiency, which impacts the normal clearance of DNTs rather than CD8^+^ or CD4^+^ T cells [37]. Zhang and coworkers also showed that DNTs can develop in athymic mice, and these DNTs exhibit more robust killing ability than those from mice with a thymus [38]. In addition, some researchers suggest that DNTs could be derived from the bone marrow [39], the appendix [40], the liver [41], the kidney [42, 43], nasal-associated lymphoid tissue(NALT) [44], or the female genital tract [45] and thus are thymus-independent. T-cell large granular lymphocyte(T-LGL) leukaemia is the most common cause of double-negative T-lymphocytosis, in which the peripheral DNTs increase a lot, accounting for 38.5%(median) of circulating lymphocytes [46]. Using a murine anaplastic large cell lymphoma (ALCL) model, they found DNTs (DN3/4 thymocytes) with aberrant TCR α/β chain could give rise to lymphoma stem cells and induce lymphoma [47]. In NP23-NHD13 double transgenic acute myelocytic leukaemia(AML) mice model, researchers verified the stemness of DNTs(DN1/2 thymocytes) [48]. These indicate a relationship between DNTs and leukemic stem cells and supply an idea for treatment of refractory leukaemia.

Based on our transcriptome data, DNTs can be divided into different subpopulations according to the distinction of phenotype and function. Using single-cell RNA sequencing, Lu Yang et al. [30] identified five naïve DNT (nDNT) subgroups from splenocytes of male C57BL/6 mice, including resting DNT (nDNT0), which was accounted for the majority (78.0%) of nDNTs, and contributed to synthetic proteins, keeping themselves alive, preparing for activation but not killing target cells; and helper DNT (nDNT1), which could secret cytokines, such as IL-17, intermediate DNT (nDNT2), cytotoxic DNT (nDNT3), as well as innate DNT (nDNT4) cells. In addition, Lu Yang et al. [30] demonstrated the transcriptome landscape of DNTs, and indicated that IKZF2 might be a distinguished transcriptional factor of DNTs. The characterization of functional DNT subsets into innate DNT, cytotoxic DNT, and helper DNT could help to better understand the function of DNT subsets, and may facilitate diagnosis and therapy. Therefore, DNTs play critical roles in the immune system, exploring functional interactions of DNTs and other immune cells is helpful for propelling clinical application in haematological malignancies.

### Functions of DNTs

#### Antitumour functions of DNT cells

**The fas/fasL pathway**

An series of lymphocytes, such as CD8^+^ T cells, [49] TILs [50], NK cells [51] and γδT cells [52], have been employed as therapeutic effector cells in adoptive cell therapy (ACT) for their tumour cytotoxic ability, including in the treatment of haematologic malignancies. Fas-dependent pathway is one of the major mechanisms of T cell-mediated effects [53]. As unconventional Treg cells, DNTs have excellent antitumour capacity in haematologic malignancies. In Young et al.’s research, infusion of either primary DNTs or cloned DNTs prevented mice from onset and progression of lymphoma and lysed A20 lymphoma cells in vitro and in vivo without causing GVHD [54, 55]. A killing assay targeting lymphoma cells with high Fas expression and low Fas expression showed that DNT-mediated anti-leukaemia activity was dependent on the Fas pathway [20, 55]. Subsequently, researchers realized that ex vivo DNT expansion was sufficient for therapeutic needs, as discovered in acute myeloid leukaemia (AML) patients who achieved complete remission [20]. Of note, DNTs are cytotoxic to primary allogeneic blasts and autologous AML blasts [20]. In a recent study, Chen showed that pancreatic cancer cells cocultured with DNTs highly expressed Fas, caspase-8 and cleaved caspase-8, which are necessary in the Fas/FasL signalling pathway [56]. Therefore, they identified a Fas-dependent mechanism underlying the anti-pancreatic cancer effect of DNTs [57].

### Innate lymphoid cell-like cytotoxic mechanisms

Innate lymphoid cells (ILCs), including NK cells, play a predominant role against cancer [58]. NK cells account for a major proportion of ILCs and act as common cytotoxic cells that induce antitumor cytotoxicity in a non-antigen-specific manner [59]. Similar to NK cells, DNTs exert innate killing ability in a non-antigen-specific manner. Using a patient-derived xenograft model of AML, Lee et al. demonstrated that DNTs showed notable antitumor effects both ex vivo and in vivo [60]. The researchers proved that DNTs encountering tumour cells expressed increased NKG2D and DNAM-1 [60], which are activating receptors and are expressed by most NK cells [61]. Binding with their ligands (MICA/B and ULBP1-6 for NKG2D and CD155 and CD112 for DNAM-1), which are expressed in tumour cells at a high level, induces effective antitumor activity [62]. Xu et al. [63] found elevated expression of NKG2D and MICA in vivo in a pancreatic carcinoma model. The researchers demonstrated a NKG2D-dependent mechanism of DNT antitumor activity [63]. Lee’s study [60] used a patient-derived AML xenograft model, and they observed high levels of NKG2D and DNAM-1, as well as their respective ligands, in DNTs and AML cells. Moreover, secretion of IFN-γ by DNTs after interaction with tumour cells massively increases the expression of ligands in AML cells and promotes killing activity [60]. In addition to NKG2D and DNAM-1, when DNTs are cocultured with haematologic malignancies cell lines, NKp30, KIR2DS4 and membrane TRAIL (mTRAIL), which are all actively involved in cytotoxicity, are also detected [64]. Interestingly, in the study, costimulatory molecules CD30, GITR, CD27 and CD28 expression of DNTs increase, while coinhibitory molecules ICOS, CTLA4, PD-1 and PD-1 ligands are low, suggesting well viability of DNTs [64].

### Cytokines

The secretion of IFN-γ, TNF, perforin and granzyme B has been discovered in haematologic malignancies models. Genetically cloned DNTs (T4H2) secrete IFN-γ and TNF upon recognizing specific antigen-loaded melanoma cells [65]. When ex vivo-expanded DNTs recognize AML cells, they express IFN-γ and TNF at levels that are similar to or higher than those seen in bulk activated CD8^+^ T cells [20], and this phenomenon was also shown after recognition of lung cancer cells by DNTs [66]. Ponzetta et al. [67] proposed a novel antitumour pathway by which tumour-associated neutrophils can induce IL-12 secretion by macrophages and thus lead to the polarization of cytotoxic DNTs, ultimately achieving tumour inhibition in an IFN-γ-dependent manner. IFN-γ has been shown to play a critical antitumour role and to participate in cancer immunosurveillance and the upregulation of cytotoxic ligands such as TNF, FasL and TRAIL [68], enhancing anti-leukaemia capacity. Additionally, the perforin-dependent pathway is strongly related to DNT-mediated antitumour activity. Ex vivo-expanded DNTs exhibit an effective ability to target and kill allogeneic blasts and autologous leukaemic blasts in a perforin- and granzyme B-dependent manner, which was confirmed in Lee’s study [20, 60]. In a study of DNT-mediated killing of melanoma cells, DNTs showed high expression of perforin and granzyme B, indicating DNT activity [65], which was consistent with research on cytotoxic DNTs targeting NSCLC cells [66]. There may be other mechanisms by which DNTs enact their antitumour abilities, and discovering the interactions of the various involved pathways will deepen our understanding of DNT cellular immunotherapy.

### Regulation of adaptive and innate immunity

Cell types with immunoregulatory functions, including CD4^+^ regulatory T (Treg) cells, CD8^+^ T cells and γδTCR^+^ T cells, play crucial roles in HSCT and tumour immunity [69]. DNTs were first demonstrated to possess regulatory functions in 2000 [15]. Accumulated evidence demonstrated that DNTs paly crucial role in both adaptive and innate immunity. Using a murine model, Zhang et al. proposed that DNTs could eliminate CD8^+^ T cells in a cell–cell contact-dependent manner [15]. The eradication of CD8^+^ T cells is Fas-dependent and antigen-specific and is closely related to trogocytosis [70], a phenomenon resulting in membrane acquisition by the majority of lymphocytes. DNTs can express MHC-peptide complexes to APCs, interact with syngenic CD8^+^ T cells through TCR-MHC recognition and subsequently kill the cells via the Fas-FasL pathway [71, 72]. In addition, Mackensen and coworkers studied human peripherally derived DNTs and found that they showed high expression of perforin and antigen-specific killing of CD4^+^/CD8^+^ T cells [65, 73]. DNTs from humans share similar regulatory functions with those from murine models [73, 74]. The cytotoxic activity of murine DNTs has also been demonstrated to be perforin- and granzyme B-dependent [34, 75]. The IL-7/Akt/mTOR signalling pathway was recently found to play a potential role in the regulatory functions of DNTs during their elimination of target cells. A recent study showed that DNTs restrain mTOR signalling in CD4^+^ T cells, resulting in the inhibition of glucose uptake, which suggests that DNTs can also suppress metabolic alterations [76]. DNTs have shown an ability to inhibit CD4^+^ and CD8^+^ T cells, and researchers have demonstrated that both ex vivo- and in vivo-induced DNTs can prevent immune rejection in an antigen-specific manner in allogeneic and xenogeneic skin and cardiac grafts in transgenic and wild-type mouse models [15, 18, 77, 78].

**Fig. 2:**
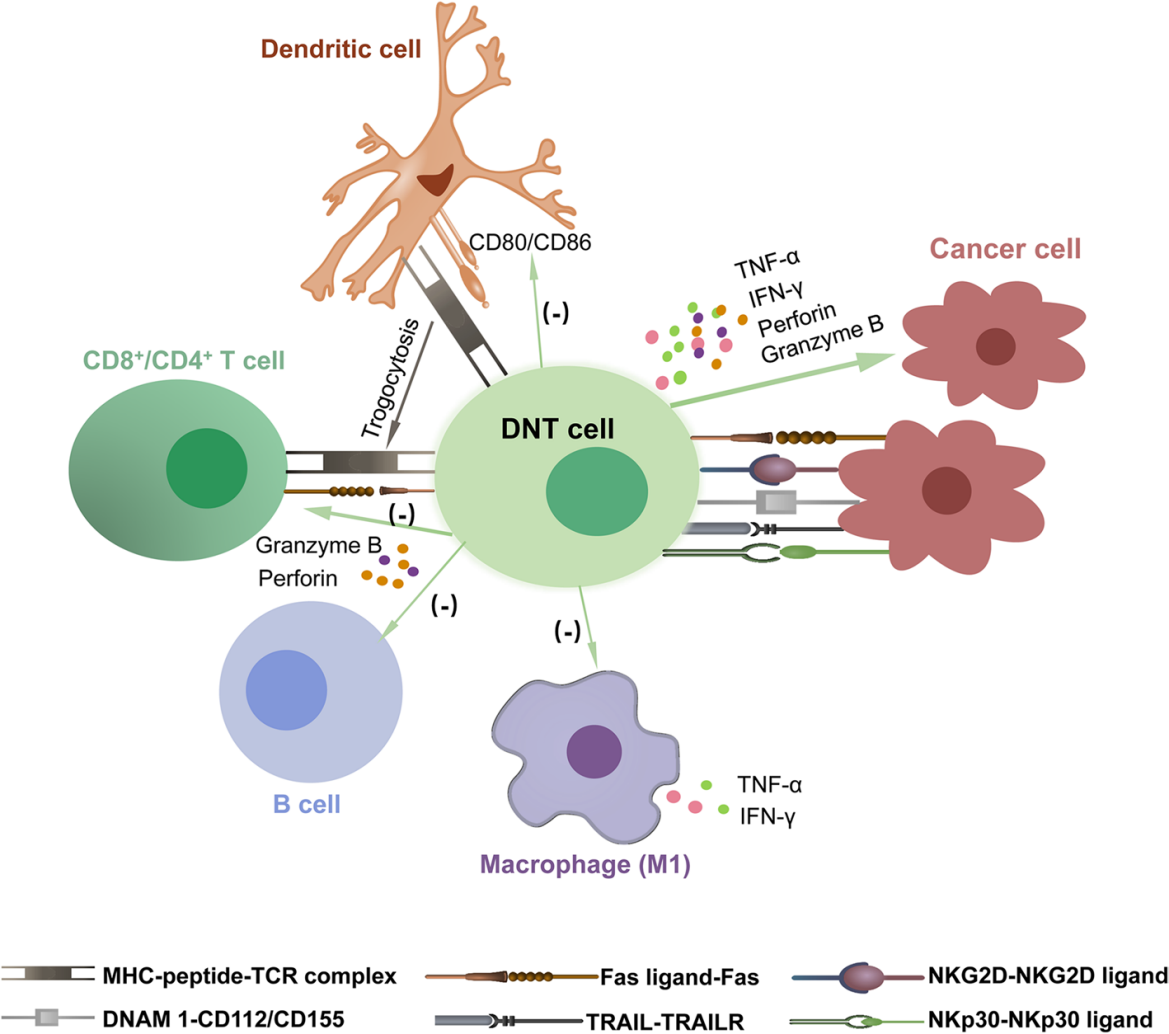
The regulatory and antitumor functions of DNTs. The antitumor functions of DNTs depend on expression of FasL, NKG2D, DNAM-1, TRAIL and NKp30. Moreover, secretion of IFN-γ, TNF-α, granzyme B and perforin take an important place. In addition, DNTs can mediate many types of immunocyte and exert immunoregulatory functions. DNTs downregulate expression of CD80/CD86 in dendritic cell and impact their interaction with other cells. DNTs can express MHC-peptide complex which expressed in dendritic cell by trogocytosis way, then interact with T cell and kill them through Fas/Fas L manner. Granzyme B and perforin secretion by DNTs induce death of T cell and B cell. Alternatively, DNTs kill M1 macrophage by down-regulating TNF-α and IFN-γ secretion of M1. FasL, Fas ligand; TNF-α, tumor necrosis factor α; NKG2D, natural killer group 2-member D; TRAIL: tumour necrosis factor-related apoptosis-inducing ligand

Of interest, accumulation of infiltrating DNTs was found in donor-specific skin grafts, and the interaction of CXCR5 expressed in DNTs and CXCL13 upregulated in cardiac graft after transplantation was essential for the migration of DNTs into the allografts [79]. What is more, CXCR5/CXCL13 interaction is important for DNTs home to lymph nodes and kidneys [29, 79, 80]. In addition, DNTs infiltrated in the allograft could kill syngeneic CD8 effector T cell and induce immune tolerance. This finding indicates that DNTs could play a role in the local graft [81]. Moreover, antibodies secreted by mature B cells hinder long-term tolerance in allografts and transplantation [82, 83]. In a xenograft model, adoptive transfer of xenoreactive DNTs lysed B cells via perforin- and granzyme B-dependent mechanisms and inhibited IgG subtypes, which resulted in alteration of rejection patterns from acute vascular rejection mainly mediated by antibodies to cell-mediated rejection [75, 84]. During this process, the TCR/MHC-peptide interaction may increase the killing activity of DNTs by effectively stimulating DNT proliferation [85]. In Hu’s study [86], DNTs interacted with B cells in a natural killer group 2-member D (NKG2D)-NKG2D ligand-dependent manner and released accumulated granzyme B, which accelerated B cell apoptosis and prevented the proliferation of B cells. Dendritic cells (DCs) plays an important role in innate immunity. Data have shown that DNTs highly expressing NKG2D tend to lyse DCs through the Fas/FasL pathway in an antigen-specific manner and downregulate the expression of CD80 and CD86 in DCs, which prevents them from interacting with T cells [87] (Fig. 2). Of note, infusion of autologous tolerogenic DCs is also able to induce immunosuppression by producing Epstein-Barr virus-induced gene 3 (EBI3), after which IFN-γ secretion by DNTs is increased [88].

Moreover, Lu Yang et al. reported that different naïve DNT subsets (resting DNT (nDNT0), helper DNT (nDNT1), intermediate DNT (nDNT2), cytotoxic DNT (nDNT3), and innate DNT (nDNT4)) also upregulated several genes associated with innate or adaptive immune responses, therefore, the functions of five identified DNT subgroups may span a wide spectrum from innate to adaptive immune responses [30].

### Clinical application of DNTs

#### Relationship with GVHD and GVL

DNTs play an important role in anti-leukaemia and regulating immune response. In Lee’s study [64], DNTs expanded under good manufacturing practice (GMP) conditions were cytotoxic to myeloma, T-cell leukaemia, Burkitt lymphoma, and AML In light of the promising regulatory function and antitumour ability of DNTs, the transfer of DNTs could result in excellent antitumor effects without causing GVHD, which is a major obstacle in cellular immunotherapy [89].

Our data showed the expanded honor-derived DNTs effectively kill leukemic cell lines and primary cells derived from leukaemia patient without toxicity to CD34^+^ hematopoietic stem cells. What is more, DNTs suppressed the proliferation of CD4^+^ T cells in vitro. A first-in human clinical trial conducted by our transplantation center found that the in vivo infusion of DNTs is safe. Through RNA-seq, we demonstrated the increased expression of *GZMA*, *GZMB* and *GZMK* in CD8^+^ T cell cocultured with DNTs. Furthermore, we used reverse transcription-polymerase chain reaction (RT-qPCR) as well as flow cytometry, and confirmed the increased expression of *GZMB* and IFN-γ of CD8^+^ T cell. Therefore, we suggest allogenetic DNTs may preserve the GVL effect by increased expression of IFN-γ and *GZM* of CD8^+^ T cells.

In Ye’s research [90], the researchers detected DNT-cell reconstitution in 73 patients undergoing allo-HSCT at days 30, 60, 90 and 180 and demonstrated delayed reconstitution in patients who developed acute GVHD. The researchers demonstrated a reverse linear relationship between the reconstitution of DNTs at 60 days and the severity of GVHD [90]. Coincidentally, Hillhouse et al. proposed that decreases in the levels of DNTs significantly affect the occurrence of chronic GVHD by mediating B cells [91]. In addition, McIver et al. [92] investigated a cohort of 40 patients experiencing GVHD after HSCT and found a negative relationship between DNT number and grade of GVHD.

#### Novel clinical therapies

DNTs are potential ACT candidates for the treatment of haematopoietic malignancies due to their substantial graft-versus-leukaemia effect and GVHD suppression. Lee et al. [64] established a novel method to produce clinical-grade therapeutic DNTs. Off-the-shelf DNTs have outstanding killing ability in various cancers without causing GVHD and remain effective after cryopreservation [64]. To explore the safety and efficacy of DNTs, a first-in-human phase I/IIa clinical trial (ChiCTR1900023499) of the infusion of unrelated third-party donor DNTs for the treatment of AML patients who relapsed after allo-HSCT conducted by our transplantation centre was completed. After lymphodepleting preconditioning, 10 AML patients with relapsed or residual disease after allo-HSCT were treated with DNTs. No patients developed GVHD or ≥ grade 3 DNT treatment-related toxicity. An initial response was observed in 70.0% of evaluable patients. Five of the DNT-treated patients remained alive, and 4 remained in complete remission (CR) for 16.5 to 24.3 months after DNT treatment. The predicted 2-year overall survival after DNT treatment was 50.0%. This study supports the safety, feasibility, and potential efficacy of allo-DNTs as a novel ACT for relapsed AML after allo-HSCT. Using CRISPR, Soares [93] screened genes in DNT treatment-resistant AML and identified the expression of CD64 as a strongly related factor that can be used as a marker to predict the effect of DNT cellular therapy. Moreover, novel treatments for adoptive DNT combined with other treatments have been explored. Chen found that infusion of DNTs after treatment with conventional chemotherapy, such as cytosine arabinoside (Arac) and daunorubicin (DNR), has greater cytotoxic activity in chemotherapy-resistant AML [94]. The same outcome was obtained when DNTs and venetoclax, a bcl-2 inhibitor, and DNTs and PD-1 inhibitors were combined [95, 96]. These findings provide suggestions for combining DNT cellular therapy with other methods for curing refractory cancer.

### Perspectives and conclusions

DNTs have been demonstrated to have great cytotoxic activity towards various haematopoietic cancers, such as myeloma, T cell leukaemia, Burkitt’s lymphoma, and AML, in vitro and to possess cytotoxicity against AML in vivo without HLA restriction [64]; in addition, they have been shown to prevent GVHD. Currently, healthy donor-derived DNTs can be expanded in vitro and maintain viability and cytotoxic ability after being cryopreserved under GMP conditions [64]. However, the characteristics and subtypes of DNTs are not fully understood.

Though DNT therapy is a novel form of adoptive cellular therapy (ACT) with promising result in treating relapsed or refractory AML in preclinical studies, the in vivo persistence and efficacy of DNTs need to be improved. Hyeonjeong Kang et al. [97] used an PI3Kδ inhibitor, idelalisib (Ide) and expanded DNTs. They verified that Ide treatment could improve the overall fitness of DNTs to promote better engraftment and longer persistence in vivo. Given that clinical application of both DNTs and Ide have been tried, application of these findings into clinic might contribute to enhancing the efficacy and durability of response [97]. What is more, DNTs therapy could significantly reduce lung tumor growth in patient-derived xenograft models but could not eradicate the full extent of the tumor cells [98].

After infusion of DNTs [60], ~ 30% of the primary AML cells remain.

DNT cells play important role both in regulatory functions and in protective immune responses, therefore, new studies are needed to elucidate mechanisms of their differentiation, regulation, effector activities, as well as the in vivo kinetics in patients. Clarifying these issues could not only provide knowledge of the pathogenic and protective mechanisms of human diseases but also further develop the potential use of DNT cells as therapeutic strategy.

## Data Availability

The datasets used and/or analyzed during the current study are available from the corresponding author on reasonable request.

## Abbreviations

HSCs: Hematopoietic stem cells
allo-HSCT: Allogeneic hematopoietic stem cell transplantation
GVHD: Graft-versus-host-disease
ACT: Adoptive cellular therapy
CAR: Chimeric antigen receptor
NK: Natural killer
TIL: Tumor-infiltrating-lymphocytes
CRS: Cytokine-release-syndrome
ICANS: Immune effector cell associated neurotoxicity syndrome
DNTs: Double negative T cells
TCR: T cell receptor
MHC: Major histocompatibility complex
DP: Double positive
SP: Single positive
T-LGL: T-cell large granular lymphocyte
ALCL: Anaplastic large cell lymphoma
IELs: Intraepithelial lymphocytes
DCs: Dendritic cells
NALT: Nasal-associated lymphoid tissue
Treg cell: Regulatory T cell
DLI: Donor lymphocyte infusion
NKG2D: Natural killer group 2-member D
EBI3: Epstein-Barr virus-induced gene 3
ILC: Innate lymphoid cell
mTRAIL: Membrane tumor necrosis factor-related apoptosis-inducing ligand
sTRAIL: Soluble tumor necrosis factor-related apoptosis-inducing ligand
NSCLC: Non-small-cell lung cancer
RT-qPCR: Reverse transcription-polymerase chain reaction
Arac: Cytosine arabinoside
DNR: Daunorubicin
Ide: Idelalisib
